# Nanoscale Inhomogeneities Mapping in Ga-Modified Arsenic Selenide Glasses

**DOI:** 10.1186/s11671-017-1887-1

**Published:** 2017-02-06

**Authors:** Ya. Shpotyuk, S. Adamiak, A. Dziedzic, J. Szlezak, W. Bochnowski, J. Cebulski

**Affiliations:** 1 Center for Innovation and Transfer of Natural Sciences and Engineering Knowledge, Faculty of Mathematics and Natural Sciences, University of Rzeszow, 1, Pigonia Str., 35-959 Rzeszow, Poland; 2 Department of Sensor and Semiconductor Electronics, Ivan Franko National University of Lviv, 107, Tarnavskoho Str., Lviv, 79017 Ukraine

**Keywords:** Arsenic selenide glass, Nanoindentation, Crystallization, Phase separation

## Abstract

Nanoscale inhomogeneities mapping in Ga-modified As_2_Se_3_ glass was utilized exploring possibilities of nanoindentation technique using a Berkovitch-type diamond tip. Structural inhomogeneities were detected in Ga_x_(As_0.40_Se_0.60_)_100−x_ alloys with more than 3 at.% of Ga. The appeared Ga_2_Se_3_ nanocrystallites were visualized in Ga-modified arsenic selenide glasses using scanning and transmission electron microscopy. The Ga additions are shown to increase nanohardness and Young’s modulus, this effect attaining an obvious bifurcation trend in crystallization-decomposed Ga_5_(As_0.40_Se_0.60_)_95_ alloy.

## Background

Chalcogenide glasses (ChG), e.g., chemical compounds of chalcogens (S, Se, or Te, but not O) with some elements from IV–V groups of the Periodic table (such as As, Sb, Ge, Bi) prepared by rapid quenching from a melt have found widespread application in modern photonics and optoelectronics because of their superior transmittance in IR domain ranged from visible to nearly 20–25 μm [1–3]. This important class of disordered materials sometimes distinguished as functional media of chalcogenide photonics [4] can be well-represented by several canonical systems (model glass-formers), where arsenic triselenide As_2_Se_3_ (i.e., As_40_Se_60_ as classified in specialized glass-chemistry terminology) in the form of melt-quenched bulky rods, drawn fibers, deposited, or sputtered thin films, etc. plays a crucial role [1–4].

For a long time, these As_2_Se_3_-type ChG have been preferentially used as passive photonics elements, only transmitting light from one point to another. In the last decades, it was shown that due to purposeful rare-earth (RE) doping, these glasses could be also employed for a number of very important active device applications [3, 4]. In this case, the mid-IR light can be initiated by emission of excited RE ions (such as Pr^3+^, Er^3+^, Dy^3+^, Tb^3+^) on different wavelengths, thus creating the remote sources of light [5–8]. From purely implementation point, it is important to achieve a high enough concentration of RE ions in ChG. One of best solutions relies on introducing Ga (or In) into ChG matrix, permitting dissolution of higher ratio of RE dopants [8–13]. However, the Ga additions may essentially restrict glass-forming ability in many ChG systems [8, 11, 13–15] provoking parasitic devitrification processes at a nanoscale through phase separation, crystallite nucleation, growth, and extraction (uncontrolled spontaneous crystallization). Thus, it was shown, that in case of glassy As_2_Se_3_ it is not possible to introduce more than 3 at.% of Ga without such intrinsic structural decomposition, which essentially influences the ChG functionality [8, 12, 14].

It is understandable that reliable experimental monitoring of such nanoscale inhomogeneities in Ga-modified ChG is very important problem in the engineering of modern chalcogenide photonics. In this work, such methodology based on nanoindentation mapping supported by a number of electron microscopy visualization techniques will be examined at the example of Ga-modified As_2_Se_3_ glasses.

## Methods

Conventional melt-quenching technique was employed to prepare Ga_x_(As_0.40_Se_0.60_)_100−x_ (x = 0_−_5) samples using high purity commercial elemental precursors of Ga (7N), As (5N), and Se (5N) [12–14]. The As and Se were specially purified by distillation with low evaporation rate to remove impurities such as oxygen, water, silica, and carbon. The appropriate amounts of initial elements with total weight of 30 g were introduced into a silica tube of 10 mm in diameter. The ampoule was sealed under vacuum and heated up to 900 °C in a rocking furnace for 10 h followed by quenching into water from 700 °C. After quenching, the samples were swiftly moved to preheated furnace for annealing for 5 h at the temperature of 10 °C below glass transition temperature (to remove mechanical strains induced by fast quenching). The obtained rods were cut into disks of ~2 mm in thickness and polished to high optical quality.

**Fig. 1:**
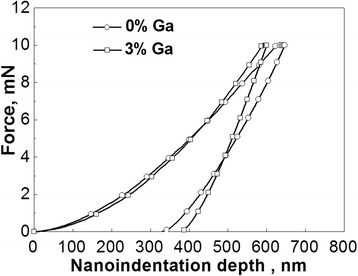
Nanoindentation load-displacement curves for As_40_Se_60_ and Ga_3_(As_0.40_Se_0.60_)_97_ ChG

The method of *nanoindentation mapping* [16] was probed as a tool to disclose possible nanoscale inhomogeneities caused by Ga additions in As_40_Se_60_ glass. The values of nanohardness (NHD) and reduced elastic modulus (the Young’s modulus *E*) were detected with a help of CSM nanoindentation instrument (CSM Instruments SA, Peseux, Switzerland) equipped with a pyramidal Berkovitch-type diamond tip with a radius of about 100 nm employing the known Oliver-Pharr method [17] for data analysis. The standard samples of fused silica with elastic modulus of 73 GPa and Poisson’s ratio of 0.17 were used for indenter calibration allowing reliable load and displacement resolution at the level of 10 nN and 0.1 nm, respectively. The trapezoidal load-displacement curves (as those shown in Fig. 1 for As_40_Se_60_ and Ga_3_(As_0.40_Se_0.60_)_97_ glasses) were detected simultaneously for maximal load of 10 mN and loading-unloading rate of 20 mN/min, the dwell time at maximal loading being set to 15 s.

The tested sample’s surface was scanned within a uniform grid of nanoindentation series (incl. 7–10 separate measurements). Such arranged experimental measuring protocol allows a quite acceptable locality of each measuring test, eliminating an influence of indentation-size effects [18–20]. The values of NHD and Young’s modulus *E* were statistically averaged for each series and a whole sample’s surface in final.

The surface morphology of fresh cut-sections of the prepared alloys was additionally visualized using scanning electron microscope (SEM) with energy-dispersive X-ray spectroscopy (EDS) analyzer FEI QUANTA 3D 200i. The transmission electron microscopy studies with primary electron beam accelerated by 200 kV voltage were performed with a FEI Tecnai Osiris device.

## Results and Discussion

**Fig. 2:**
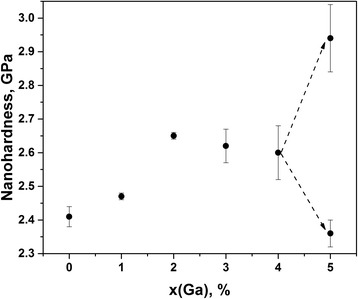
Compositional mapping curves for nanohardness NHD in Ga_x_(As_0.40_Se_0.60_)_100−x_ alloys

In respect to our previous research on melt-quenched alloys in Ga_x_(As_0.40_Se_0.60_)_100−x_ system [8, 12, 14], the phase decomposition processes accompanied by Ga_2_Se_3_ crystallization occur at 4 at.% of Ga. Thus, glasses with no more than 3 at.% of Ga were suggested to be successfully used for further RE doping [8]. The results of nanoindentation mapping presented on Fig. 2 confirm the homogeneity in these ChG via NHD and Young’s modulus *E* measurements. With Ga content, both of these parameters show eventual growing tendency as it is character for other homogeneous glassy alloys affected by dopants which increase density.

**Fig. 3:**
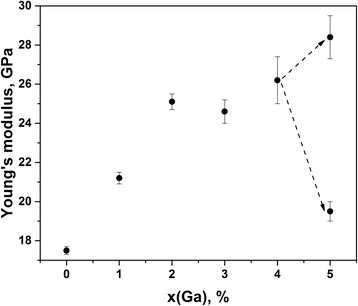
Compositional mapping curves for Young’s modulus *E* in Ga_x_(As_0.40_Se_0.60_)_100−x_ alloys

It is known that structural evolution in glassy As_40_Se_60_ at small amount of Ga added [14] is preferentially governed by appearance of As_2_Se_4/2_ blocks based on homonuclear As–As covalent chemical bonds in a glassy network, which overbalance Ga-centered polyhedral units (GaSe_4/2_ tetrahedrons). Such processes occur under growing input of atomic-deficient volumes contributing from bond-free solid angles around neighboring Se atoms terminated As_2_Se_4/2_ fragments. This void agglomeration trend is quickly saturated with Ga additions in ChG, thus facilitating mechanical freedom for gathering of Ga-based units. The partially decomposed Ga_4_(As_0.40_Se_0.60_)_96_ alloy display an obvious increase of scattering in the NHD (Fig. 2) and Young’s modulus *E* (Fig. 3) values. The drastic changes in the nanoindentation mapping are character for higher Ga content, just in Ga_5_(As_0.40_Se_0.60_)_95_ alloy. Both the NHD and *E* parameters are essentially bifurcated in multiple measuring series of indentation testing. The down level of bifurcation is more or less tightly grouped around some averaged values (NHD = 2.36 GPa and *E* = 19.5 GPa), which are very close to those observed in As_40_Se_60_ glass, while the upper level is more roughly shifted, especially for Young’s modulus *E*. Noteworthy, with activation of crystallization in Ga_4_(As_0.40_Se_0.60_)_96_ and Ga_5_(As_0.40_Se_0.60_)_95_ alloys, the character of free-volume void evolution is drastically changed, and now void fragmentation prevails due to stabilizing relaxation of growing Ga_2_Se_3_ crystallites [14].

The surface morphology of fresh cut-sections of these alloys were visualized with the help of electron microscopy to identify the appeared Ga_2_Se_3_ crystallites.

**Fig. 4:**
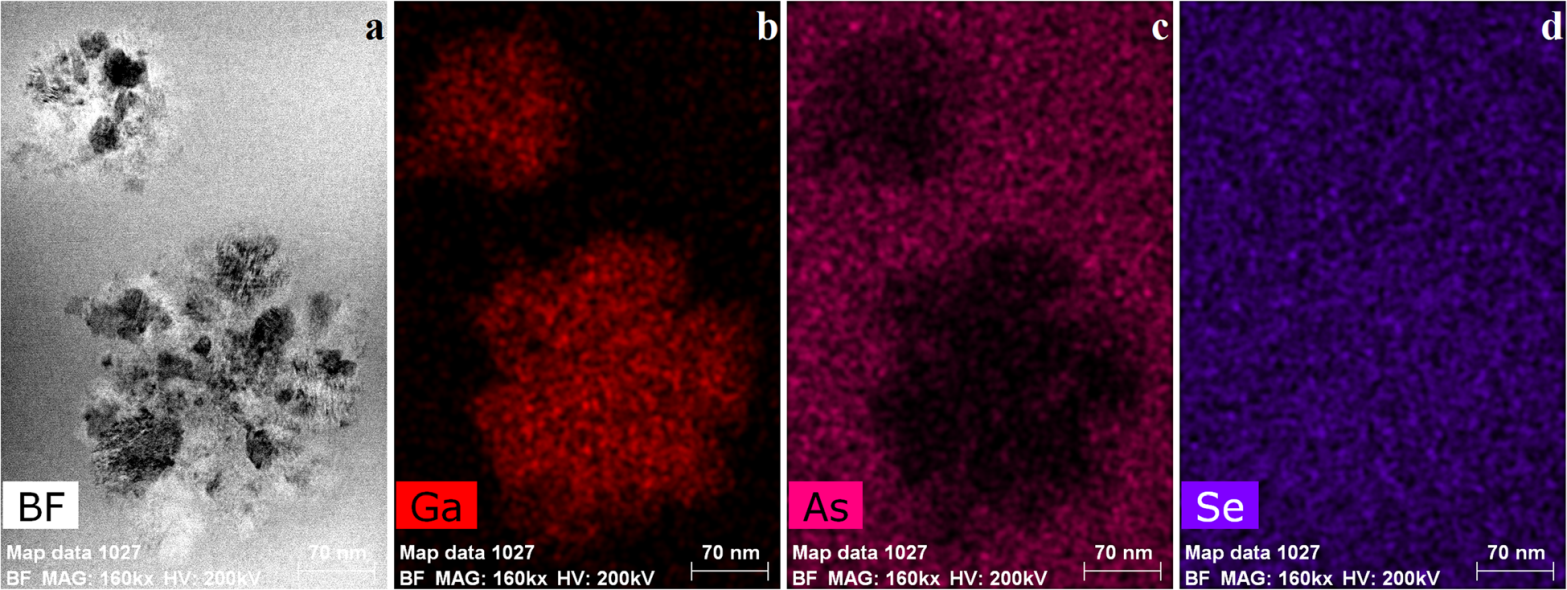
Microstructure cut-section view of Ga_4_(As_0.40_Se_0.60_)_96_ alloy in STEM Bright Field (**a**), and chemical composition EDS mapping of Ga (**b**), As (**c**), and Se (**d**)

In Ga_4_(As_0.40_Se_0.60_)_96_ alloy, these crystallites represent an agglomeration of tightly connected separate pieces extended over 200–300 nm (see Fig. 4a). The EDS spectroscopy performed in scattered electrons of all constituting elements (Fig. 4b–d) allows reliable identification of crystallites composition, i.e., Ga_2_Se_3_ which is in excellent respect to the XRD data [8, 14]. Such crystallites cannot essentially affect nanoindentation mapping over a whole sample’s surface because of comparative sizes with indenter imprints, but they eventually enhance scattering in the NHD and Young’s modulus *E* values.

**Fig. 5:**
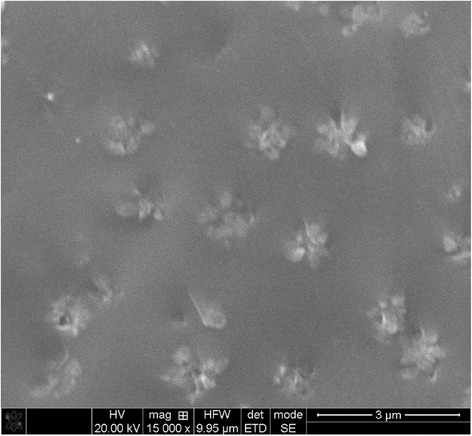
SEM micrograph of freshly prepared cut-section of Ga_5_(As_0.40_Se_0.60_)_95_ alloy showing nearly uniform random distribution of flower-like Ga_2_Se_3_ crystallite inclusions

In contrast, the Ga_2_Se_3_ crystallites in Ga_5_(As_0.40_Se_0.60_)_95_ alloy get grown to larger μm sizes (Fig. 5). Under nearly uniform random distribution of such flower-like Ga_2_Se_3_ crystallite agglomerates (as it shown in Fig. 5), they left large spaces of ChG in a homogeneous glassy state. Of course, this glass is slightly enriched on As, but still close to undoped stoichiometric As_2_Se_3_. As a result, the bifurcation effect is observed in nanoindentation mapping, giving two groups of NHD and Young’s modulus *E* data.

## Conclusions

Mapping of nanoscale inhomogeneities in Ga-modified As_2_Se_3_ glassy alloy was utilized exploring possibilities of conventional nanoindentation technique equipped with a Berkovitch-type diamond tip. Structural inhomogeneities were detected in Ga_x_(As_0.40_Se_0.60_)_100−x_ alloys having more than 3 at.% of Ga. The appearance of Ga_2_Se_3_ nanocrystallites was separately visualized in Ga-modified arsenic selenide glass using scanning and transmission electron microscopy. The Ga additions are shown to increase nanohardness and Young’s modulus of glasses, this effect attaining an obvious bifurcation trend in crystallization-decomposed Ga_5_(As_0.40_Se_0.60_)_95_ alloy.

## Abbreviations

ChG: Chalcogenide glass
EDS: Energy-dispersive X-ray spectroscopy
NHD: Nanohardness
RE: Rare-earth
SEM: Scanning electron microscope
